# Nondipping blood pressure pattern predicts cardiovascular events and mortality in patients with atherosclerotic peripheral vascular disease

**DOI:** 10.1177/1358863X231161655

**Published:** 2023-04-10

**Authors:** Nina Dahle, Johan Ärnlöv, Jerzy Leppert, Pär Hedberg

**Affiliations:** 1 Center for Clinical Research Dalarna, Uppsala University, Falun, Sweden; 2 Primary Health Care Center Britsarvet-Grycksbo, County of Dalarna, Falun, Sweden; 3 Department of Neurobiology, Division of Family Medicine and Primary Care, Care Sciences and Society, Karolinska Institutet, Huddinge, Sweden; 4 School of Health and Social Studies, Dalarna University, Falun, Sweden; 5 Center for Clinical Research, Uppsala University, Västmanland County Hospital, Västerås, Sweden; 6 Department of Clinical Physiology, Västmanland County Hospital, Västerås, Sweden

**Keywords:** ambulatory blood pressure monitoring, cardiovascular risk prediction, peripheral vascular disease, peripheral artery disease (PAD)

## Abstract

**Background:**

Patients with peripheral vascular disease (PVD) are often underdiagnosed and undertreated. Nocturnal nondipping blood pressure (BP) pattern, as diagnosed by ambulatory BP monitoring (ABPM), is associated with increased cardiovascular risk, but has not been studied in patients with PVD. We aimed to investigate if a nondipping BP pattern predicts cardiovascular events or all-cause death in outpatients with PVD.

**Methods:**

Consecutive outpatients with carotid or lower-extremity PVD were examined with 24-hour ABPM (*n* = 396). Nondipping was defined as a < 10% fall in systolic BP level during night-time. We used Cox regression models adjusting for potential confounders. We also evaluated the incremental prognostic value of dipping status in the COPART risk score. Our primary composite outcome was cardiovascular events or all-cause death.

**Results:**

In the cohort (mean age 70; 40% women), 137 events occurred during a 5.1-year median follow-up; incident rate of 7.35 events per 100 person-years. Nondipping was significantly associated with outcome (hazard ratio 1.55, 95% CI 1.07–2.26, *p* = 0.021) in a fully adjusted model. When adding nondipping to the risk markers in the COPART risk score, the model fit significantly improved (χ^2^ 7.91, *p* < 0.005) and the C-statistic increased from 0.65 to 0.67.

**Conclusion:**

In a cohort of outpatients with PVD, nondipping was an independent risk factor for future cardiovascular events or mortality and seemed to be a strong predictor in patients with carotid artery disease but not in lower-extremity PVD. Additional studies are needed to evaluate the clinical utility of ABPM for improved prevention in these high-risk patients. **(ClinicalTrials.gov Identifier: NCT01452165)**

## Background

Atherosclerotic peripheral vascular disease (PVD) is a common disease defined as systemic arterial atherosclerosis outside the aorta and coronary and intracranial arteries.^1,2^ Intermittent claudication is the most recognized symptom, but most patients with PVD are asymptomatic. Nevertheless, even asymptomatic patients with PVD face an increased risk of major cardiovascular events and mortality.^1,3^ PVD is considered a coronary heart disease risk equivalent.^4^ However, patients with PVD remain underdiagnosed and undertreated in primary care, where disease awareness is low despite a high PVD prevalence.^1,3,5^ Previously, we found that most PVD patients had poorly controlled ambulatory blood pressure (BP) profiles and suboptimal control of other important cardiovascular risk factors,^6^ suggesting that better preventive strategies are needed in this high-risk group.^7^

BP is a leading risk factor for cardiovascular disease in the general population^8^ and patients with PVD.^1^ BP is a dynamic parameter characterized by complex variability affected by environmental, physical, and emotional factors.^9^ Ambulatory BP monitoring (ABPM) provides the average of BP readings over a defined period, usually 24 hours, and provides the opportunity to assess nocturnal values.^10,11^ The BP normally decreases 10–20% during sleep. More than a 10% fall in nocturnal BP is defined as normal dipping, whereas a < 10% reduction in BP is defined as nondipping.^11^ The term nondipping was first introduced in 1988 when O’Brien et al. reported that a less marked decrease in nocturnal BP led to a greater prevalence of stroke.^12^ It is now established that nondippers have a substantially increased cardiovascular risk.^13^ Nocturnal BP is recognized to be superior to daytime BP in predicting cardiovascular risk.^10,14,15^ The nondipping BP pattern is strongly associated with prognostic outcomes in patients with uncomplicated hypertension and the general population.^16–18^ To our knowledge, the prognostic significance of nondipping in patients with PVD has not been reported.

We hypothesized that nondipping is a relevant prognostic marker for an increased risk for cardiovascular events also in patients with PVD. We aimed to investigate the association between nondipping and the incidence of cardiovascular events or all-cause mortality during a long-term follow-up of outpatients with confirmed carotid or lower-extremity PVD.

## Methods

### Study population

Analyses were based on patients included in the Peripheral Arterial Disease in Västmanland study (PADVa), based on patients with atherosclerotic PVD.^19^ All patients visiting the ultrasound laboratory of the Department of Vascular Surgery at the Västmanland County Hospital in Västerås, Sweden, between April 2006 and February 2011, were considered for inclusion. Causes of referral were claudication (45%), transient ischemic attack or stroke (26%), aortic aneurysm (8%), heart murmur (5%), suspected renal artery stenosis/renovascular hypertension (4%), and others (12%). Every patient was examined with ultrasonography to identify any stenosis in the internal carotid artery (ICA). Patients with claudication symptoms also underwent ankle BP measurement, to calculate the ankle–brachial index (ABI), and ultrasonography of the arteries in the symptomatic leg. The patients were invited to participate in the PADVa study if they fulfilled at least one of the following inclusion criteria: (i) mild to severe stenosis or occlusion of the ICA; (ii) symptoms of claudication combined with ABI ⩽ 0.90 in the symptomatic lower extremity; or (iii) symptoms of claudication combined with ultrasonographic evidence of arterial occlusive disease in the same extremity.

In total, 452 patients (73.6%) accepted the invitation to join the study. Everyone in the study was offered ABPM, of whom 35 individuals declined. We excluded patients with < 10 daytime or < five night-time ABPM measurements (*n =* 15)^20^ and missing values on blood analyses (*n* = 6), leaving 396 patients for the present analysis.

The study was approved by the Ethics Committee of Uppsala University, Sweden (Dnr 2005:382). All participants gave their written informed consent. The study is identified as ClinicalTrials.gov Identifier NCT01452165.

### Examination protocol

All patients were invited to attend the Department of Clinical Physiology and were examined according to a standard examination protocol, including an extensive self-administered questionnaire including smoking status (current smoking defined as regular smoking within the past year), medical history, and current medication. Self-reported diagnoses of cardiovascular disease and diabetes mellitus were confirmed from the medical records. Hypertension was defined as present if diagnosed by a physician and treated with antihypertensive medication.

### Blood samples

Participants fasted overnight, and venous blood samples were taken by trained staff and immediately sent to the accredited Laboratory of Clinical Chemistry, Västmanland County Hospital, Västerås. The estimated glomerular filtration rate (eGFR) was calculated from serum creatinine levels standardized by isotope dilution mass spectrometry (Synchron LX or UniCel DxC instruments; Beckman Coulter Inc., Brea, CA, USA) using the chronic kidney disease epidemiology (CKD-EPI) formula.^21^ Serum total cholesterol concentration was determined using a UniCel DxC 800 or Synchron LX20 Analyzer (Beckman Coulter Inc.). In addition, blood samples were frozen at −70°C within 2 hours. In 2017, high-sensitivity C-reactive protein (hs-CRP) was analyzed on thawed samples using the Cobas C501 instrument (Roche Diagnostics, Basel, Switzerland).

### Ankle–brachial index and carotid ultrasound

Blood pressure in both arms and ankles was measured in all participants in a supine position after at least 5 minutes of rest. The ankle BP was measured in the bilateral dorsalis pedis and posterior tibial arteries using an inflatable leg-cuff, an aneroid sphygmomanometer, and a handheld Doppler instrument with a 5-MHz probe. The ankle–brachial index (ABI) was calculated by dividing the highest ankle pressure by the highest BP of both arms. An ABI of ⩽ 0.90 or ⩾ 1.40 in either leg was defined as abnormal. Ultrasound examinations of the carotid and lower-limb arteries have been described in detail.^19^ Briefly, grading of ICA lesions into a normal artery, plaque without flow disturbance, mild/moderate/severe stenosis, or occlusion was based on grey-scale images, color flow Doppler scans, and spectral Doppler blood flow velocities (online Supplementary Table 1S).^22^

### Clinical and ambulatory blood pressure

Office BP was measured manually by trained technicians and obtained from the nondominant arm or the other arm if the systolic BP was > 10 mmHg higher. The BP was measured in the supine position after a minimum of 5 minutes of rest and was rounded up to the nearest 2 mmHg. Using the arm from which the office BP was obtained, the ABPM-04 instrument (Meditech Ltd, Budapest, Hungary) was applied for 24-hour ABPM, with readings taken every 20 minutes.^23^ Three different cuff sizes were available and selected depending on the patient’s upper arm size. Day- and night-time periods were assessed from the time of awakening and sleeping entered by the patient in a diary card.

Nondipping was defined as a reduction in night-time systolic BP ⩽ 10% of the daytime average systolic BP and dipping as a nightly fall by more than 10% of the daytime average systolic BP value.^13,24^

### Outcomes

The participants were followed through the Swedish population and in-patient registries until a cardiovascular endpoint, all-cause death, or December 31, 2013, at which time the remaining participants were censored. The cardiovascular endpoints were defined as hospitalization or death caused by myocardial infarction (International Classification of Diseases 10th Revision [ICD-10] code I21), stroke (ICD-10 I61 or I63), and heart failure event (ICD-10 I11.0, I25.5, or I50). As endpoints, we also included hospitalization because of percutaneous coronary intervention or coronary artery bypass grafting.

### Statistics

Baseline data are presented as mean ± SD or frequency and percentage. Highly skewed variables (hs-CRP) are presented as median (25^th^, 75^th^ percentiles). Statistical comparisons between groups were made using the unpaired *t*-test for continuous variables with normal distribution, the Kruskal–Wallis test in highly skewed continuous variables (hs-CRP), and the χ^2^ test for categorical variables. To investigate the association of nondipping with cardiovascular events or mortality, we used Cox regression in four separate multivariable models. Model A included age and sex. Model B included age, sex, and office and ambulatory 24-hour systolic and diastolic BP. Model C adjusted for age, sex, ambulatory 24-hour systolic and diastolic BP, and other potential confounders (total cholesterol, body mass index [BMI], smoking status, eGFR, diabetes, hypertension, heart failure, previous myocardial infarction, previous stroke, ICA stenosis, and abnormal ABI). Model D included the six variables from the validated COhorte de Patients ARTériopathes (COPART) risk score: age, history of myocardial infarction, eGFR, ABI, hs-CRP, and medication with renin-angiotensin system inhibitors, statins, and antiplatelet agents.^25,26^ The goodness of fit between two nested Cox models was compared with the likelihood-ratio test. The proportional hazard assumption was verified visually by scaled Schoenfield residuals. The discriminatory performance of the models (i.e., the ability of a model to differentiate between high- and low-risk patients) was assessed by C-statistics.

In sensitivity analyses, we performed weighted Cox regression based on the propensity score to balance the baseline characteristics of the dippers and nondippers. We performed these analyses in the entire study cohort and in subgroups with lower extremity PVD and carotid artery disease, respectively. The propensity score was obtained by multiple logistic regression, including all variables from Table 1. From the propensity score, weights were obtained by calculating stabilized inverse probability treatment weights (IPTWs).^27^ The groups were considered balanced when the absolute standardized mean differences between all baseline variables were < 10%. The IPTWs were subsequently used in weighted Cox regression models with dipping status as an independent variable. If a baseline variable was impossible to balance between groups, the variable was also included as an independent variable in the weighted Cox regression model to reduce residual confounding.

**Table 1.** Baseline characteristics of the study cohort.

	All *n* = 396	Dippers *n* = 211	Nondippers *n* = 185	*p*-value
Female sex	158 (39.9%)	78 (37.0%)	80 (43.2%)	0.242
Age, years	70.0 ± 7.0	68.9 ± 6.9	71.3 ± 7.0	< 0.001
Current smoking	62 (15.7%)	42 (19.9%)	20 (10.8%)	0.019
BMI, kg/m^2^	27.1 ± 4.2	26.7 ± 3.9	27.6 ± 4.4	0.031
Office systolic BP, mmHg	154 ± 21	152 ± 21	155 ± 20	0.101
Office diastolic BP, mmHg	77 ± 10	76 ± 9	77 ± 10	0.200
24-h amb systolic BP, mmHg	131 ± 14	129 ± 13	133 ± 14	0.015
24-h amb diastolic BP, mmHg	68 ± 9	68 ± 8	67 ± 9	0.252
Diabetes	95 (24.0%)	39 (18.5%)	56 (30.3%)	0.009
**Medical history**				
Hypertension	306 (77.3%)	146 (69.2%)	160 (86.5%)	< 0.001
Heart failure	29 (7.3%)	9 (4.3%)	20 (10.9%)	0.021
Previous myocardial infarction	75 (18.9%)	33 (15.6%)	42 (22.7%)	0.097
Previous stroke	42 (10.6%)	17 (8.1%)	25 (13.5%)	0.111
Total cholesterol, mmol/L	4.6 ± 1.2	4.5 ± 1.0	4.6 ± 1.3	0.234
Abnormal ABI	221 (55.8%)	107 (50.7%)	114 (61.6%)	0.038
ICA stenosis	298 (75.3%)	150 (71.1%)	148 (80.0%)	0.053
ICA stenosis and abnormal ABI	131 (33.1%)	53 (25.1%)	78 (42.2%)	< 0.001
hs-CRP, mg/L	2.10 (1.00, 4.43)	1.80 (0.90, 4.05)	2.50 (1.10, 4.90)	0.012^a^
eGFR, mL/min per 1.73 m^2^	73.7 ± 20.2	75.3 ± 18.9	71.9 ± 21.6	0.101
**Medication at discharge**				
COPART medication^b^	160 (40.4%)	76 (36.0%)	84 (45.4%)	0.072
Beta-blocker	198 (50.0%)	85 (40.3%)	113 (61.1%)	< 0.001
ARB	93 (23.5%)	45 (21.3%)	48 (25.9%)	0.336
ACE-I	138 (34.8%)	66 (31.3%)	72 (38.9%)	0.137
Aspirin	308 (77.8%)	165 (78.2%)	143 (77.3%)	0.925
Diuretic	94 (23.7%)	41 (19.4%)	53 (28.6%)	0.042
Statin	323 (81.6%)	172 (81.5%)	151 (81.6%)	> 0.999

Values are mean ± SD, median (25th, 75th percentiles) or *n* (percentage).

a Kruskal–Wallis test *p*-value.

b Medication as defined in the COPART risk score (i.e., aspirin, ACE-I, ARB, or statins).

Amb, ambulatory; ABI, ankle–brachial index; ACE-I, angiotensin converting enzyme inhibitor; amb, ambulatory; ARB, angiotensin receptor blocker; BMI, body mass index; BP, blood pressure; eGFR, estimated glomerular filtration rate; hs-CRP, high-sensitivity C-reactive protein; ICA, internal carotid artery.

Statistical analyses were performed using SPSS statistical software for Windows, version 26 (IBM, Armonk, NY, USA) and R 3.5.3 (R Foundation for Statistical Computing, Vienna, Austria). Two-sided *p*-values less than 0.05 were considered to be statistically significant.

## Results

### Baseline characteristics

The baseline characteristics of the patients, in total and divided into dippers and nondippers, are presented in Table 1. Ambulatory systolic BP was higher among nondippers, whereas the two groups were comparable in diastolic BP levels. The nondippers were moderately older, with slightly higher BMI. Fewer participants with nondipping were smokers (11%) compared to dippers (20%). Diabetes (30% vs 18%) and hypertension (86% vs 69%) were more prevalent in nondippers than dippers. Heart failure was more than twice as common in nondippers compared with dippers (11% vs 4%), whereas no significant differences in the prevalence of previous myocardial infarction or stroke were detected. Levels of total cholesterol were similar between the groups, but hs-CRP was higher among nondippers. Cardiovascular medications included in the COPART risk score (antiplatelet agents, statins, and renin-angiotensin inhibitors) were similarly used among the groups. Diuretics and beta-blockers were more frequently used among nondippers.

### Cardiovascular events

**Figure 1.**
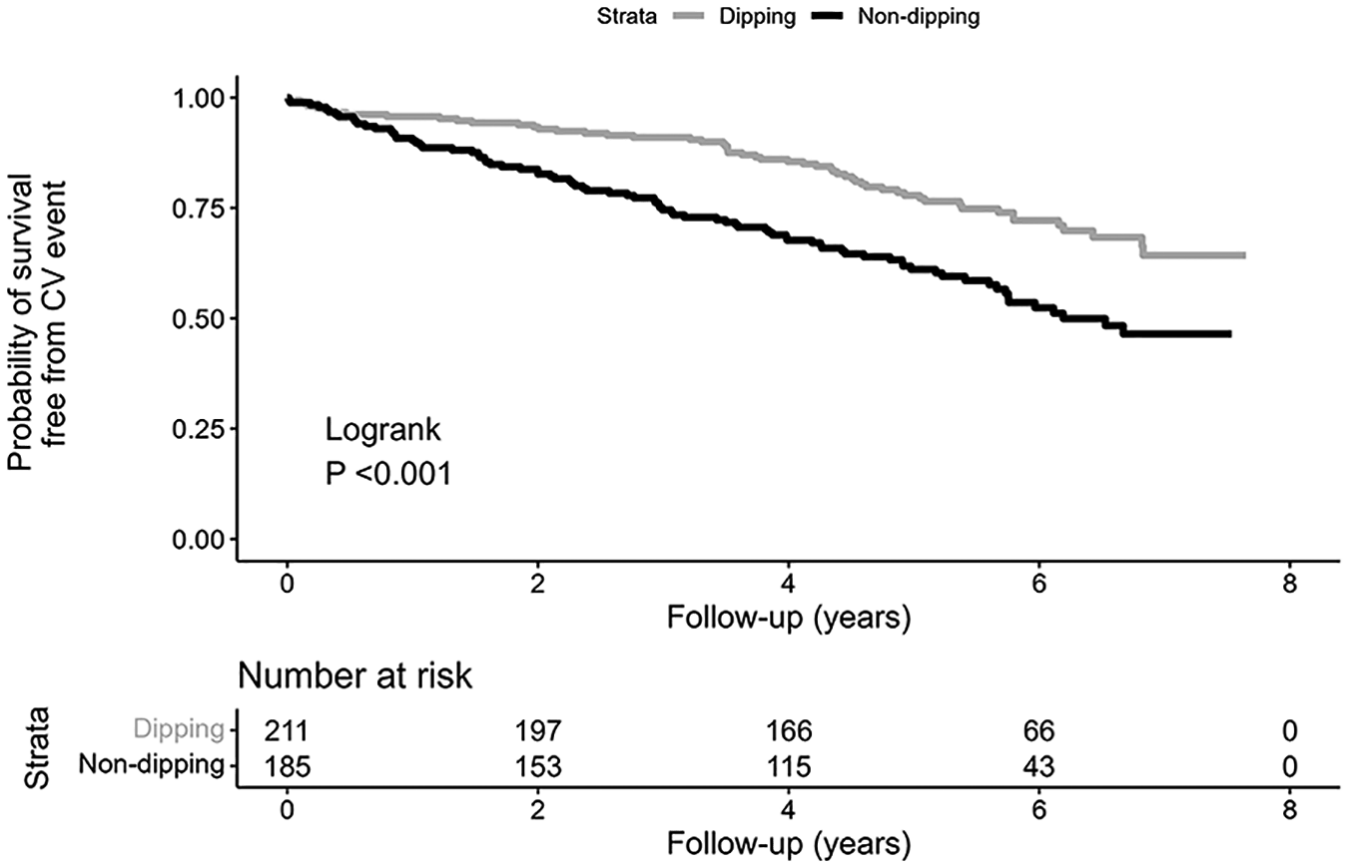
Kaplan–Meier curve displaying survival free from cardiovascular event in dippers and nondippers.

The number of incident cardiovascular events during follow-up with numbers at risk and incidence rates among the participants are presented in Table 2. A Kaplan–Meier curve depicts the probabilities of survival free from cardiovascular disease in nondippers and dippers (Figure 1).

**Table 2.** Risk and incidence of events in the whole cohort and by dipping status.

Events	Whole cohort (*n* = 396)		Dipping (*n* = 211)		Nondipping (*n* = 185)	
	No. of events	Incidence rate (per 100 PYAR)	No. of events	Incidence rate (per 100 PYAR)	No. of events	Incidence rate (per 100 PYAR)
Myocardial infarction or coronary intervention	53	2.8	26	2.4	27	3.4
Heart failure	27	1.4	10	0.9	17	2.1
Stroke	29	1.6	7	0.7	22	2.7
All-cause mortality	65	3.5	22	2.1	43	5.3
Cardiovascular event or all-cause mortality	137	7.4	55	5.2	82	10.2

PYAR, person-years at risk.

### Cox regression analyses

Nondipping was significantly associated with adverse outcomes in all multivariable models, with a hazard ratio (HR) of 1.55 (95% CI 1.07–2.26, *p* = 0.021) compared with dipping when adjusting for all potential confounders (Table 3). The model fit significantly improved when dipping status was included in the COPART risk score (likelihood-ratio test χ^2^ 7.91, *p* < 0.005). The C-statistic estimate increased from 0.65 to 0.67.

**Table 3.** Risk of cardiovascular events and all-cause mortality in nondippers versus dippers (multivariable Cox regression).

	Dipping	Nondipping	*p*-value
	HR	HR (95% CI)	
Unadjusted	1.00 (ref.)	1.99 (1.41–2.80)	< 0.001
Model A^a^	1.00 (ref.)	1.92 (1.36–2.73)	< 0.001
Model B^a^	1.00 (ref.)	1.95 (1.36–2.79)	< 0.001
Model C^a^	1.00 (ref.)	1.55 (1.07–2.26)	0.021
Model D^a^	1.00 (ref.)	1.65 (1.16–2.35)	0.005

Based on the full study cohort (*n* = 396) and 137 events.

a Model A adjusted for age and sex. Model B adjusted for age, sex, office and ambulatory 24-hour systolic and diastolic blood pressures. Model C adjusted for age, sex, ambulatory 24-hour systolic and diastolic blood pressures, total cholesterol, BMI, smoking, diabetes, hypertension, previous myocardial infarction, previous stroke, heart failure, eGFR, ABI, and internal carotid stenosis. Model D adjusted for variables included in the COPART risk score, i.e., age, previous myocardial infarction, hs-CRP, ABI, eGFR, and medication with statins, aspirin, and angiotensin receptor blocker.

ABI, ankle–brachial index; BMI, body mass index; eGFR, estimated glomerular filtration rate; hs-CRP, high-sensitivity C-reactive protein.

### Sensitivity analyses

In sensitivity analyses, we reached a satisfactory balance of baseline characteristics between dippers and nondippers in the weighting based on propensity score in the entire study cohort (*n* = 396; online Supplementary Table 2S) and in the subgroup of patients with ICA stenosis only (*n* = 167; online Supplementary Table 3S). In contrast, in the patients with abnormal ABI only (*n* = 90), the weighting failed to reach a balance for sex, age, total cholesterol, and hs-CRP (online Supplementary Table 4S). Therefore, we included these variables in the subsequent weighted Cox regression to reduce the possibility of residual confounding.

In the weighted Cox regression, nondipping was significantly associated with outcome in the whole cohort (HR 1.45, 95% CI 1.04–2.03, *p* = 0.031). In the subgroup with ICA stenosis (41 events), the association of nondipping with outcome demonstrated an HR of 2.49 (95% CI 1.30–4.76, *p* = 0.006). In the patients with abnormal ABI only (32 events), nondipping was not significantly associated with adverse outcomes (HR 1.03, 95% CI 0.50–2.11, *p* = 0.936).

## Discussion

### Principal findings

In the present sample of outpatients with PVD, nondipping was an independent predictor of cardiovascular events or all-cause death. Nondipping remained significantly associated with prognosis when adjusting for age, sex, 24-hour BP levels, and established cardiovascular risk factors. Dipping status also improved risk prediction when added to the validated COPART risk score. Our findings show that the previously reported association between nondipping and adverse events in the general population also holds for patients with PVD.^16–18^ Interestingly, our subgroup analyses suggested that nondipping was strongly associated with adverse outcomes in patients with carotid artery disease but not in patients with lower extremity PVD.

### Comparison with the literature

The results that nondipping is a risk factor for adverse prognosis in patients with PVD is in line with studies in hypertensive patients and in the general population, as well as in patients in hemodialysis.^16–18,28^ Our findings that the association between nondipping and outcome was independent of mean systolic and diastolic 24-hour BP levels, are in accordance with previous findings in hypertensive patients.^16^

The prevalence of nondipping is higher in high-risk cohorts; for example, in patients with diabetes and renal disease. As expected, the prevalence of nondipping was markedly higher (43%) in our cohort compared to a general hypertensive population (25%).^29^

Smoking is a particularly strong risk factor for PVD, and a higher proportion of the participants in this study were smokers than the general population in Sweden.^6^ Notably, fewer nondippers were smokers compared to the dippers, which might result from reverse causality bias, where subjects with a higher burden of cardiovascular disease have stopped smoking as secondary prevention.

Interestingly, nondipping was associated considerably stronger with incident adverse events in our subgroup with carotid artery disease than in the subgroup with abnormal ABI. Previous data suggest that the ABPM nondipping pattern is more strongly associated with incident stroke events than with cardiac events.^30^ Given the known increased risk of stroke associated with carotid artery disease,^31^ we can only speculate that stroke events might drive the difference between our subgroups. These findings merit further studies.

### Potential mechanisms

The pathogenetic mechanism behind nondipping and cardiovascular disease is incompletely understood. However, it is believed to have multiple causes, such as activity during the day, the depth and quality of sleep, and activity of the sympathetic nervous system, among others.^29^ One possible mechanism linking night-time BP with cardiovascular disease is that nondipping could be associated with elevated circulating levels of molecules related to endothelial dysfunction and atherosclerosis.^32^ Nondippers have previously been found to be more likely to have aortic stiffness,^33,34^ and arterial stiffness is a characteristic for patients with PVD.^35^ Another possible pathophysiological mechanism is that renal dysfunction may induce a higher night-time BP to sustain natriuresis,^36^ a mechanism supported by the slightly lower renal function in our nondipping cohort.

Obstructive sleep apnea is associated with a significantly increased risk of nondipping.^37^ Sleep apnea is a highly prevalent chronic condition in the general population, characterized by recurrent nocturnal desaturation episodes inducing sympathetic activity and often associated with hypertension, obesity, diabetes, and metabolic syndrome.^38^ Since we did not have any data on sleep apnea in our cohort, it was not possible to investigate this issue further.

### Clinical relevance

A previous study suggests that bedtime antihypertensive drug administration can partially restore a normal dipping pattern.^39^ A multicenter, controlled, prospective trial found that bedtime hypertension treatment in over 19,000 hypertensive patients decreased BP during sleep and increased dipping, which improved cardiovascular risk reduction with a diminished occurrence of major cardiovascular disease events.^40^ It is possible that bedtime administration of antihypertensive medication could benefit patients with PVD, but further studies are needed to investigate this. Better risk stratification in this high-risk group might become more important as efficient and expensive lipid-lowering PCSK9 inhibitors are becoming more widely used.^41^

The COPART risk score is a validated risk score for patients with PVD that is evaluated for long-term prediction of all cause and CV mortality.^26^ When we adjusted for the risk markers included in the COPART risk model, nondipping remained a significant predictor, and the C-statistic was slightly higher when nondipping was added to the model, indicating that there might be some benefit in using dipping status in risk stratification in patients with PVD.

### Strengths and limitations

Strengths of the study include the well-characterized study participants, the long-term follow-up, and the clinically generalizable study population of heterogeneous outpatients with carotid and/or lower-extremity PVD, both symptomatic and asymptomatic.

Limitations include that the study population was limited to outpatients of European origin who were found to have lower-extremity and/or carotid artery disease at a visit to a vascular ultrasound laboratory. The invited patients who declined to join the study (*n* = 162) did not differ in age (*p* = 0.68) or sex (*p* = 0.93) compared with the participants. However, if more burdened with disease, these dropouts may have been a source of bias. Data on medication were based on self-reported information, which can be a cause of informational bias. However, good agreement between patient interviews and computerized pharmacy records has been found in the elderly population.^42^ We do not have data on sleep quality during the ABPM, which is a limitation since sleep quality can affect nocturnal BP.^43,44^ However, the patients recorded their own sleeping hours. Further limitations include that we did not evaluate subgroups such as extreme dippers or reverse dippers since we did not have enough power for those analyses and that we only performed one measurement with ABPM and, therefore, could not investigate how reproducible the results are. Previous studies have shown poor reproducibility in nondipping.^44,45^ The COPART risk score is based on hospitalized severe PVD^25^ and is not intended for the less severe PVD in our cohort. However, to our knowledge, it is currently the only validated risk score available in this patient group.

## Conclusions

A nondipping 24-hour BP pattern was associated with an increased risk of cardiovascular events and mortality in outpatients with PVD. Nondipping is an independent risk factor for adverse outcomes, and this association might be more potent in patients with ICA stenosis than in patients with lower-extremity PVD. Further studies are needed to evaluate if a nocturnal BP profile can be used clinically to improve risk prediction and to investigate if treatment of nocturnal BP can diminish nondipping and improve prognosis.

## Supplemental Material

sj-pdf-1-vmj-10.1177_1358863X231161655 – Supplemental material for Nondipping blood pressure pattern predicts cardiovascular events and mortality in patients with atherosclerotic peripheral vascular disease Click here for additional data file. Supplemental material, sj-pdf-1-vmj-10.1177_1358863X231161655 for Nondipping blood pressure pattern predicts cardiovascular events and mortality in patients with atherosclerotic peripheral vascular disease by Nina Dahle, Johan Ärnlöv, Jerzy Leppert and Pär Hedberg in Vascular Medicine
