# Supplementary material for: Nondipping blood pressure pattern predicts cardiovascular events and mortality in patients with atherosclerotic peripheral vascular disease
Source: Vasc Med. 2023 Apr 10;28(4):274–81. doi: 10.1177/1358863X231161655 (PMC10408241; doi:10.1177/1358863X231161655)
Supplement: sj-pdf-1-vmj-10.1177_1358863X231161655 – Supplemental material for Nondipping blood pressure pattern predicts cardiovascular events and mortality in patients with atherosclerotic peripheral vascular disease [file sj-pdf-1-vmj-10.1177_1358863X231161655.pdf]

Supplemental Material

Table 1S. Ultrasonographic grading of internal carotid artery lesions in the PADVa study.

Grade	Plaque	Color flow Doppler	Spectral Doppler
Normal artery	Absent	No turbulence	No spectral broadening<br>Systolic Vmax < 1.2 m/s
Plaque without flow disturbance	Present	No turbulence	No spectral broadening<br>Systolic Vmax < 1.2 m/s
Mild stenosis	Present	Turbulence	Spectral broadening<br>Systolic Vmax 1.2–1.4 m/s
Moderate stenosis	Present	Turbulence	Spectral broadening<br>Systolic Vmax 1.5–2.5 m/s
Severe stenosis	Present	Turbulence	Spectral broadening<br>Systolic Vmax > 2.5 m/s
Occlusion	Present	No flow	No flow

Vmax indicates maximum flow velocity.

Table 2S. Baseline characteristics of the complete study cohort (n = 396) before and after inverse probability treatment weighting (IPTW) based on propensity score.

	Unweighted sample			Weighted sample		
	Dippers n = 211	Non-dippers n = 185	Diff.*	Dippers	Non-dippers	Diff.*
Female sex	78 (37.0%)	80 (43.2%)	0.062	40.0%	40.1%	0.005
Age (years)	68.9 ± 6.9	71.3 ± 7.0	0.356	70.2 ± 6.5	70.7 ± 7.6	0.073
Current smoking	42 (19.9%)	20 (10.8%)	-0.252	15.3%	14.6%	-0.007
BMI (kg/m2)	26.7 ± 3.9	27.6 ± 4.4	0.224	27.1 ± 4.2	27.1 ± 4.4	-0.002
24-h amb systolic BP (mmHg)	129 ± 13	133 ± 14	0.251	131 ± 14	131 ± 14	0.047
24-h amb diastolic BP (mmHg)	68 ± 8	67 ± 9	-0.119	68 ± 8	68 ± 9	-0.028
Diabetes	39 (18.5%)	56 (30.3%)	0.120	23.6%	24.7%	0.011
**Medical history**						
Hypertension	146 (69.2%)	160 (86.5%)	0.172	77.3%	82.0%	0.047
Heart failure	9 (4.3%)	20 (10.9%)	0.066	7.2%	7.4%	0.002
Previous myocardial infarction	33 (15.6%)	42 (22.7%)	0.072	16.6%	18.4	0.018
Previous stroke	17 (8.1%)	25 (13.5%)	0.055	10.3%	10.2%	-0.001
Total cholesterol (mmol/L)	4.5 ± 1.0	4.6 ± 1.3	0.113	4.5 ± 1.1	4.5 ± 1.2	0.005
Abnormal ABI	107 (50.7%)	114 (61.6%)	0.107	54.5%	54.6%	0.001
ICA stenosis	150 (71.1%)	148 (80.0%)	0.088	76.8%	77.4%	0.006
Log-hs-CRP (mg/L)	0.64 ± 1.07	0.91 ± 1.10	0.245	0.80 ± 1.14	0.77 ± 1.14	-0.031
eGFR (mL/min per 1.73 m2)	75.3 ± 18.9	71.9 ± 21.6	-0.164	74.4 ± 19.6	73.9 ± 21.7	-0.027
**Medication at discharge**						
Betablocker	85 (40.3%)	113 (61.1%)	0.211	49.7%	51.3%	0.016
ARB	45 (21.3%)	48 (25.9%)	0.048	23.0%	24.4%	0.015
ACE-I	66 (31.3%)	72 (38.9%)	0.073	35.7%	36.9%	0.011
Aspirin	165 (78.2%)	143 (77.3%)	-0.005	75.0%	77.8%	0.028
Diuretic	41 (19.4%)	53 (28.6%)	0.094	22.3%	25.6%	0.033
Statin	172 (81.5%)	151 (81.6%)	0.005	83.6%	83.9%	0.002

Values are mean ± standard deviation or number (percentage).

Abbreviations: ABI, ankle brachial index; ACE-I, angiotensin converting enzyme inhibitor; amb, ambulatory; ARB, Angiotensin receptor blocker; BMI, body mass index; BP, blood pressure; eGFR, estimated glomerular filtration rate; hsCRP, high sensitive C-reactive protein; ICA, internal carotid artery.

*Standardized mean difference (continuous variables) or difference in proportions (categorical variables) between dippers and non-dippers.

Table 3S. Baseline characteristics of the patients with ICA stenosis only (n = 167) before and after inverse probability treatment weighting (IPTW) based on propensity score.

	Unweighted sample			Weighted sample		
	Dippers n = 211	Non-dippers n = 185	Diff.*	Dippers	Non-dippers	Diff.*
Female sex	40 (41.2%)	32 (45.7%)	0.045	42.3%	43.3%	0.001
Age (years)	68.6 ± 6.7	71.0 ± 7.6	0.328	69.6 ± 6.6	70.3 ± 7.8	0.096
Current smoking	18 (18.6%)	5 (7.1%)	-0.114	13.5%	9.9%	-0.036
BMI (kg/m2)	27.6 ± 3.7	27.7 ± 4.9	0.006	27.7 ± 3.6	27.7 ± 4.8	-0.015
24-h amb systolic BP (mmHg)	129 ± 12	133 ± 14	0.314	130 ± 13	131 ± 14	0.053
24-h amb diastolic BP (mmHg)	69 ± 9	69 ± 10	0.040	68 ± 9	68 ± 10	-0.005
Diabetes	15 (15.5%)	16 (22.9%)	0.074	18.2%	20.7%	0.025
**Medical history**						
Hypertension	67 (69.1%)	63 (90.0%)	0.209	78.2%	85.9%	0.077
Heart failure	3 (3.1%)	5 (7.1%)	0.040	3.7%	4.8%	0.011
Previous myocardial infarction	17 (17.5%)	13 (18.6%)	0.011	16.5%	15.2%	-0.013
Previous stroke	12 (12.4%)	9 (12.9%)	0.005	12.6%	11.7%	-0.009
Total cholesterol (mmol/L)	4.4 ± 0.9	4.8 ± 1.3	0.327	4.5 ± 1.0	4.5 ± 1.2	0.006
Log-hs-CRP (mg/L)	0.43 ± 0.90	0.75 ± 1.10	0.323	0.54 ± 0.90	0.57 ± 1.11	0.032
eGFR (mL/min per 1.73 m2)	76.8 ± 16.1	75.1 ± 23.6	-0.083	76.7 ± 17.1	76.6 ± 23.3	-0.005
**Medication at discharge**						
Betablocker	41 (42.3%)	36 (51.4%)	0.092	45.4%	49.1%	0.037
ARB	20 (20.6%)	19 (27.1%)	0.065	24.5%	25.5%	0.010
ACE-I	29 (29.9%)	23 (32.9%)	0.030	30.5%	33.3%	0.028
Aspirin	74 (76.3%)	49 (70.0%)	-0.063	74.8%	72.4%	-0.024
Diuretic	16 (16.5%)	18 (25.7%)	0.092	20.9%	22.5%	0.016
Statin	86 (88.7%)	56 (80.0%)	-0.087	87.3%	85.2%	-0.021

Values are mean ± standard deviation or number (percentage).

Abbreviations: ACE-I, angiotensin converting enzyme inhibitor; amb, ambulatory; ARB, Angiotensin receptor blocker; BMI, body mass index; BP, blood pressure; eGFR, estimated glomerular filtration rate; hsCRP, high sensitive C-reactive protein.

*Standardized mean difference (continuous variables) or difference in proportions (categorical variables) between dippers and non-dippers.

Table 4S. Baseline characteristics of the patients with abnormal ankle-brachial index only (n = 90) before and after inverse probability treatment weighting (IPTW) based on propensity score.

	Unweighted sample			Weighted sample		
	**Dippers n = 211**	**Non-dippers n = 185**	**Diff.***	**Dippers**	**Non-dippers**	**Diff.***
Female sex	19 (35.2%)	16 (44.4%)	0.093	38.1%	51.3%	0.132
Age (years)	67.0 ± 7.2	71.1 ± 7.5	0.558	67.9 ± 7.1	69.3 ± 7.9	0.180
Current smoking	12 (22.2%)	4 (11.1%)	-0.111	19.3%	16.3%	-0.029
BMI (kg/m2)	25.3 ± 4.2	28.1 ± 4.2	0.651	26.2 ± 4.6	26.2 ± 4.7	0.002
24-h amb systolic BP (mmHg)	127 ± 14	132 ± 14	0.389	128 ± 15	127 ± 15	-0.070
24-h amb diastolic BP (mmHg)	71 ± 9	70 ± 9	-0.137	71 ± 9	70 ± 8	-0.083
Diabetes	6 (11.1%)	10 (27.8%)	0.167	11.9%	15.0%	0.031
**Medical history**						
Hypertension	35 (64.8%)	24 (66.7%)	0.019	69.5%	76.2%	0.068
Heart failure	2 (3.7%)	3 (8.3%)	0.046	3.4%	4.2%	0.009
Previous myocardial infarction	5 (9.3%)	5 (13.9%)	0.046	8.8%	8.0%	-0.008
Previous stroke	0 (0.0%)	6 (16.7%)	0.167	0.0%	6.6%	0.066
Total cholesterol (mmol/L)	4.7 ± 1.2	4.6 ± 1.0	-0.091	4.7 ± 1.2	4.5 ± 1.0	-0.180
Log-hs-CRP (mg/L)	0.81 ± 1.08	0.99 ± 1.10	0.640	0.85 ± 1.0	0.71 ± 1.23	-0.280
eGFR (mL/min per 1.73 m2)	78.3 ± 20.5	70.4 ± 14.9	-0.442	75.6 ± 19.5	74.7 ± 15.8	-0.049
**Medication at discharge**						
Betablocker	13 (24.1%)	18 (50.0%)	0.259	26.6%	31.3%	0.046
ARB	9 (16.7%)	8 (22.2%)	0.056	20.2%	22.4%	0.022
ACE-I	15 (27.8%)	13 (36.1%)	0.083	29.4%	27.9%	-0.015
Aspirin	41 (75.9%)	31 (86.1%)	0.102	80.8%	89.5%	0.088
Diuretic	8 (14.8%)	8 (22.2%)	0.074	18.7%	18.7%	-0.001
Statin	38 (70.4%)	28 (77.8%)	0.074	73.6%	81.6%	0.080

Values are mean ± standard deviation or number (percentage).

Abbreviations: ACE-I, angiotensin converting enzyme inhibitor; amb, ambulatory; ARB, Angiotensin receptor blocker; BMI, body mass index; BP, blood pressure; eGFR, estimated glomerular filtration rate; hsCRP, high sensitive C-reactive protein.

*Standardized mean difference (continuous variables) or difference in proportions (categorical variables) between dippers and non-dippers.
